# Effects of water levels on plant traits and nitrogen use efficiency in monoculture and intercropped artificial grasslands

**DOI:** 10.3389/fpls.2022.958852

**Published:** 2022-07-27

**Authors:** Zhiqiang Wan, Rui Gu, Yulong Yan, Lijun Bai, Tiejun Bao, Jie Yang, Qingzhu Gao, Hasbagan Ganjurjav, Guozheng Hu, Haijun Zhou, Xi Chun

**Affiliations:** ^1^College of Geographical Science, Inner Mongolia Normal University, Hohhot, China; ^2^Key Laboratory of Mongolian Plateau’s Climate System, Hohhot, China; ^3^Inner Mongolia Autonomous Region Wetland Restoration Engineering Autonomous Region, Hohhot, China; ^4^College of Grassland, Resources and Environment, Inner Mongolia Agricultural University, Hohhot, China; ^5^College of Ecology and Environment, Inner Mongolia University, Hohhot, China; ^6^Ministry of Education Key Laboratory of Ecology and Resource Use of the Mongolian Plateau, Inner Mongolia University, Hohhot, China; ^7^Inner Mongolia Key Laboratory of Grassland Ecology, School of Ecology and Environment, Inner Mongolia University, Hohhot, China; ^8^Inner Mongolia Environmental Monitoring Center Station, Hohhot, China; ^9^Key Laboratory for Agro-Environment & Climate Change of Ministry of Agriculture, Institute of Environment and Sustainable Development in Agriculture, Chinese Academy of Agricultural Sciences, Beijing, China

**Keywords:** forages, efficiency, productivity, nutrient, monoculture, intercropping, artificial grassland

## Abstract

Water availability is the main factor affecting the forage productivity of artificial grasslands, particularly in semi-arid regions. Generally, intercropping of gramineous grass and leguminous grass can achieve high productivity. However, how different water availability levels affect the productivity of intercropping system remains unclear. Here, we conducted a 3-year (2015–2017) study by manipulating the water conditions (CK equivalent to the annual precipitation, +50% treatment equivalent to 50% increase over the average precipitation, and −50% treatment equivalent to 50% decrease over the average precipitation) to explore the responses of plant traits, nitrogen use efficiency, and biomass of the monoculture of *Medicago sativa* (a leguminous grass, *M.s*), monoculture of *Elymus nutans* (a gramineous grass, *E.n*), and intercropping of *M.s* and *E.n* in a semi-arid region in Inner Mongolia, China. The results showed that the biomass obtained by intercropping of *M.s* and *E.n* decreased by 24.4% in −50% treatment compared to the CK treatment, while that of the monoculture of *M.s* decreased by 34.4% under the −50% treatment compared to the CK treatment. However, there was no significant difference in the biomass between intercropping artificial grassland and monoculture *M. sativa* under +50% treatment. Compared to monoculture, M.s can obtain more nitrogen by biological nitrogen fixation and decrease the proportion of nitrogen absorbed from soils under intercropping in the same water conditions. Under the intercropping system, the proportions of nitrogen absorbed from soils by M.s were 87.4%, 85.1, and 76.9% in −50%, CK, and +50% treatments, respectively. Under monoculture, these proportions were 91.9, 89.3, and 82.3% in −50%, CK, and +50% treatments, respectively. Plant trait, but not soil nitrogen content, was the main regulator for the productivity responses to water level changes. Our results highlight that intercropping can achieve higher productivity in both dry and wet conditions. Therefore, considering the fluctuating rainfall events in the future, it might be useful to alter the proportions of intercropped forage species in an artificial grassland to obtain optimal productivity by reducing the limitations of nitrogen availability. However, the economic viability of intercropping *M. sativa* and *E. nutans* should be evaluated in the future.

## Introduction

The typical steppe plays an important role in maintaining the balance and stability of regional ecosystems in Inner Mongolia (Zhang, 2000; Bai et al., 2016). Unfortunately, in the past half-century, the steppe in this area has been severely degraded, which has restricted the sustainable development of the grassland animal husbandry industry. The appropriate establishment of artificial grasslands can alleviate the low level of productivity caused by natural grassland degradation and promote the restoration of degraded natural grasslands (Ren et al., 2016). The main forages used for artificial cultivation are the members of the Gramineae and Leguminosae families, which can be grown as monocultures or polycultures (e.g., by employing the technique of intercropping) (Xia et al., 2009; Li et al., 2016), and the primary limiting factors for the establishment of artificial grasslands in the region are water and nitrogen. An optimal level of rainfall enhances the productivity of artificial grasslands (Ren et al., 2017; Zhou et al., 2017), while a shortage of water causes wilting and a decrease in productivity (Guo et al., 2017), and excessive precipitation results in leaching of soil nitrogen, which reduces the available nitrogen in the soil (Zhu et al., 2011; Shi et al., 2018).

Additionally, plants adapt to their environment by adjusting their growth patterns and resource allocation. Specific leaf area (SLA) and stem/leaf ratio of forage, as well as the ability of plants to obtain resources, increase when water is sufficient (Knops and Reinhart, 2000; Zhu et al., 2010; Huo et al., 2013). However, some studies have shown that the stem/leaf ratio in *Medicago sativa* increased when water was insufficient or deficient. Therefore, an increase in the water content could result in a change in the nitrogen sources for forages.

Leguminous grasses fix nitrogen present in the air, which facilitates not only the meeting of their own growth needs but also the absorption and utilization of nitrogen by adjacent grasses (Meziane and Shipley, 1999; Rasmussen et al., 2007; Pirhofer-Walzl et al., 2012). Intercropping can enable such competitive absorption, which can result in an increase in nitrogenase activity, thus improving the nitrogen fixation efficiency of the legumes (Schipanski and Drinkwater, 2012). Droughts severely weaken the biological nitrogen fixation capacity of legumes (Wang et al., 2007; Guan et al., 2014). The nitrogen fixation capacity of legumes is not only affected by soil water but also by soil nitrogen content. The result showed that the nitrogen fixation capacity of legumes is significantly less when grown in soil with a higher inorganic nitrogen content (Schipanski et al., 2010). Although an increase in water content may be conducive to the growth of forages and nitrogen fixation of legumes, it also leads to the leaching of a large amount of nitrate-nitrogen (Chen et al., 2007; He et al., 2013). Studies on grassland and forest ecosystems had found that higher water use efficiency (WUE) is always associated with lower nitrogen use efficiency (NUE) (Field et al., 1983; Chen et al., 2005). There is a compensation mechanism between them. The source of nitrogen utilization and resource utilization strategies of different plants were different. WUE and NUE are not simply a trade-off relationship but interact on each other in different forages and different environment conditions. Therefore, the change in soil nitrogen content is based on the varying nitrogen consumption and nitrogen fixation functions of different forages.

The growth of forages is directly affected by water levels, which impact soil nitrogen loss and accumulation and further affect the growth of forages. Based on the previous research, we made the following hypothesis: (1) intercropped cultures of *Medicago sativa* and *Elymus nutans* was more conducive to reduce the absorption of soil nitrogen; and (2) the relationship between environmental factors, nitrogen use efficiency, and forage productivity would be different under different planting conditions. We expect to clarify (1) the forage traits and productivity in different water levels; (2) N resource of grasses and biological nitrogen fixation of *Medicago sativa* in monoculture under different water levels; and (3) the relationship between productivity and nitrogen utilization under different water levels. Therefore, understanding the impact of varying levels of water on productivity and nitrogen utilization of these plants is of great scientific significance to the construction of artificial grasslands and the efficient utilization of water and nitrogen in Inner Mongolia.

## Materials and methods

### Study area

The study was conducted in the Grassland Ecology Research Base of Inner Mongolia University, MaoDeng pasture, 35 km east of Xilinhot City, Inner Mongolia. The geographical location is 116°29′E, 44°09′N, and 1,102 m.a.s.l. The annual mean temperature is 2.6°C, and the annual average rainfall is 315 mm with the most rainfall occurring from June to September. The coldest month is January, with an average temperature of −23.5°C, while the hottest month is July, with an average temperature of 25.2°C.

### Experimental design

**FIGURE 1:**
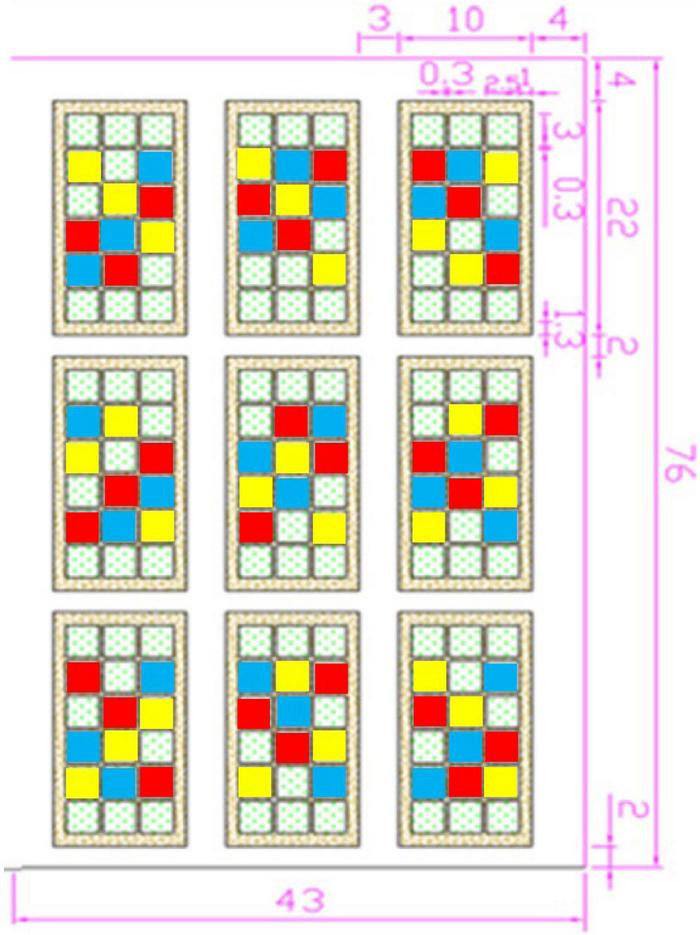
Experimental plot (dotted square: other forages planted were not studied in this paper; blue square: monocultures of *Medicago sativa*; red square: monocultures of *Elymus nutans*; and yellow square: E.n and M.s intercropped).

This experiment was carried out by using the existing automatic canopy (top open when not raining and top closed during rain, with the following awning specifications: shelter width 10.0 m, length 22.0 m, top height 3.4 m, and shoulder height 1.8 m) in the grassland ecology research base of Inner Mongolia University and mimicking the climate characteristics of a typical steppe, such as a large warming range, unobvious changes in precipitation, and large interannual fluctuations. The automatic canopy provided shelter and quantitative irrigation that allowed us to simulate different precipitation conditions found in drought and semi-drought regions (Figure 1).

**FIGURE 2:**
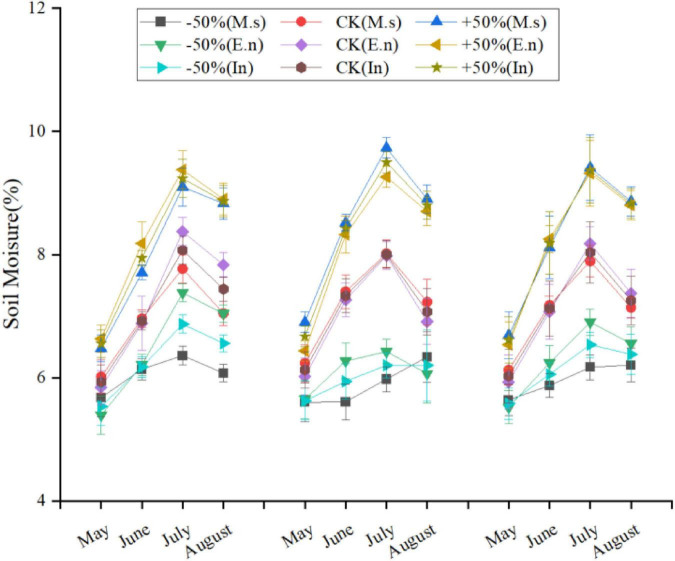
Soil moisture in May to August from 2015 to 2017 under different water levels.

According to the standard of the China Meteorological Administration, precipitation only includes vertical precipitation, such as rain, snow, hail, etc. We set different water levels according to the observed precipitation data. In the typical steppe in Inner Mongolia, the annual average precipitation during 1960–2015 was approximately 2,37.0 mm, with a maximum of 455.2 mm and a minimum of 108.7 mm, during the growing season (May to August). The control (CK) treatment was set as the multi-year average of 240 mm (equivalent to the precipitation in the growing season), the +50% treatment had increased irrigation (360 mm, equivalent to a 50% increase over the average 240 mm precipitation in the growing season), and the −50% treatment had decreased irrigation (120 mm, equivalent to a 50% decrease in the average 240 mm precipitation in the growing season). The specific irrigation amounts are listed in Table 1. The results showed that the soil moisture was significantly different under different water levels (Figure 2). Each treatment was repeated three times; accordingly, nine automatic shelters were used for the study. Eighteen plots (2.5 m × 3 m) were included in every shelter. Monocultures of *Elymus nutans* (represented as E.n) and *M. sativa* (represented as M.s) along with intercropped cultures of E.n and M.s (represented as In-E.n and In-M.s, respectively) were selected as the research objects. Six rows were planted in each sample plot, and every row was 30 cm apart. E.n and M.s were intercropped in the same row. About 30 g of seeds was planted in every plot, with 15 g of *M. sativa* seeds and 15 g of *E. nutans* seeds intercropped.

**TABLE 1:** Irrigation amounts for each treatment group corresponding to the average rainfall for each month of the growing season from 1960 to 2015.

Month	1960–2015 Average rainfall (mm)	Irrigation amount (L⋅m^–2^)		
		–50%	+50%	CK
5	25.4	3.2	9.5	6.4
6	47.3	5.9	17.7	11.8
7	79.7	10.0	29.9	19.9
8	62.6	7.8	23.5	15.6

### Measurement indices and methods

#### Environmental factors

Soil moisture levels at 5 cm depth were measured using temperature and humidity recorders. The observation frequency was 20 min.

### Growth and functional traits of forage

Plant height: Six plants with uniform growth were selected and marked in each plot, and the plant height was measured using a meter ruler in August every year. The height of *M. sativa* was taken as the distance from the base of the stem to the top of the leaf. The height of *E. nutans* was recorded as the height of the vegetative branch.

Leaf area: Ten healthy herbage plants with uniform growth and wide leaves were selected in each plot, and 50 leaves were randomly selected and measured using a Li-3000c leaf area meter.

Leaves and stems were placed in envelopes, dried at 65°C in an oven, and then weighed.


(1) SLA=leafarea/dryleafweight

(2) Stem/leafratio=drystemweight/dryleafweight

Aboveground biomass: 1 m × 3 m quadrats with uniform growth (stubble height = 3–5 cm) in each plot were selected. The fresh weight of all forages in each quadrat was measured, after which the plants were dried at 65°C in an oven to constant weight, and the dry weights were measured.

### Nitrogen content

Soil samples at a depth of 0–70 cm were collected from each experimental plot in August 2015 and 2016 and were divided into five layers: 0–10, 10–20, 20–30, 30–50, and 50–70 cm. Five soil cores were randomly sampled in each plot using an auger (3.8 cm in diameter and 10 cm in depth) and were mixed together as one composite sample. Soil samples were passed through a 2-mm diameter soil sieve, and visible living plant roots were discarded before analyses. The samples were stored in self-sealed bags, allowed to thaw, and then sifted before analysis. Fresh soil samples were extracted with 0.5 mol/L KCl (1:4, soil: extractant), and NH_4_^+^-N and NO_3_^–^-N levels were determined using an auto-analyzer (Bran+Luebbe Auto Analyzer 3, Germany). The total soil nitrogen content was analyzed using an elemental analyzer after air-drying the samples. The moisture content of fresh soil was 10.82% under −50%, 13.78% under CK, and 15.68% under +50%.

### Nitrogen use efficiency of plants

Plant samples were collected from the plots in August 2015 and 2016. The plant samples were dried, sieved, and placed in tin cups. The nitrogen content was analyzed using an elemental analyzer.

Nitrogen use efficiency (NUE) of forage: C/N of a leaf was used to express the long-term NUE of the plants (Chapin and Cleve, 2000; Livingston et al., 2010; Zhan et al., 2012).

### Determination of nitrogen fixation and nitrogen transfer

The ^15^N-labeled fertilizer [(^15^NH_4_)_2_SO_4_, abundance was 98.4%] solution prepared in 9L of distilled water was applied evenly in each plot using a spray bottle, which is equivalent to 1.5 mm of rainfall. The amount of fertilizer applied was equivalent to 0.03 g N/m^2^, which was far less than the soil nitrogen content and would not affect soil processes or stimulate plant growth. To avoid the tracer being absorbed by the leaf surface, an additional amount of water was sprayed after the tracer application. The total volume of the distilled water used to spray equaled 3 mm of rainfall.

Plant samples were collected and divided into roots, stems, and leaves within the range of 0.5 m × 0.5 m in the marked center area in July after 2 months of tracing and then soaked in 0.5 mol/L of CaCl_2_ solution for 30 min to remove the residual N on the leaf surfaces. Afterward, they were washed with distilled water, placed in envelopes, and dried at 75°C. Soil samples were collected at the same time and were divided into five layers: 0–10, 10–20, 20–30, 30–50, and 50–70 cm.

The soil and plant samples were ground into powder (particle size of approximately 5 μm) by using a mixed ball mill (MM400, Retsh) and passed through a 0.125-mm diameter sieve. These samples were analyzed using a MAT253 stable isotope mass spectrometer (Finnigan MAT, Bremen, Germany).

(1) The formulas used to calculate biological nitrogen fixation rate (P_*ndfa*_) and biological nitrogen fixation capacity (W_*ndfa*_) are as follows:


(2) In-P(%)n⁢d⁢f⁢a=(1-A%⁢E⁢of⁢In⁢-⁢M.sA%⁢E⁢of⁢E.n)×100

(3) $$M.s\text{-}P_{n\,d\,f\,a}\left(\%\right)=\left(1-\frac{A\%\,E\,\mathrm{of}\,M.s}{A\%\,E\,\mathrm{of}\,E.n}\right)\times 100$$

Wndfa (g N/m^2^⋅a) = P_*ndfa*_ × total nitrogen yield of leguminous forage.

A%E is the abundance of ^15^N in plants.

(2) Of the total plant nitrogen (P_*ndfl*_), the percentage of nitrogen that In-E.n obtained from nitrogen fixed by In-M.s and the nitrogen transfer amount (W_*ndfl*_) were calculated as follows:


(4) $${\text{P}}_{\text{ndfl}}\left(\%\right)=\left(1-\frac{A\%\,E\,\mathrm{of}\,\mathrm{In}\,\text{-}\,E.n}{A\%\,E\,\mathrm{of}\,E.n}\right)\times 100$$

(5) $${\text{W}}_{\text{ndfl}}\,\left(\mathrm{gN}/m^{2}\cdot a\right)={\text{P}}_{\text{ndfl}}\times \mathrm{total}\,\mathrm{nitrogen}\,\mathrm{yield}\,\mathrm{of}\,\mathrm{In}\,\text{-}\,E.n$$

(3) Of the total plant nitrogen, the percentage of N absorbed by *M. sativa* and *E. nutans* from the soil was calculated as follows:


(6) $$\%\mathrm{Ndfs}\left(E.n\right)=1-P_{\mathrm{ndfl}}\left(\%\right)$$

(7) $$\%\mathrm{Ndfs}\left(\mathrm{In}\text{-}M.s\right)=1-\mathrm{In}\text{-}P_{\mathrm{ndfa}}\left(\%\right)$$

(8) $$\%\mathrm{Ndfs}\left(M.s\right)=1-M.s\text{-}P_{\mathrm{ndfl}}\left(\%\right)$$

(4) The increased rate of nitrogen fixation of In-M.s caused by the transfer of nitrogen fixation products in intercropping (with M.s as the reference) was calculated as follows:


(9) %Ndfai=(1-A%EofIn-M.s/A%EofM.s)×100%

(5) The contribution rate of biological nitrogen fixation to grassland nitrogen yield was calculated as follows:


Contributionrateofbiologicalnitrogenfixation(%)=

(10) $$\,W_{\mathrm{ndfa}}\times 100/\mathrm{total}\,\mathrm{nitrogen}\,\mathrm{yield}\,\mathrm{of}\,\mathrm{grassland}$$

### Data analysis

Excel 2016 was used for preliminary data arrangement. ANOVA was used for single-factor analysis to show the significance of indexes under different water levels by SPSS 19.0 (SPSS Inc., Chicago, IL, United States). Linear regression analysis of biological nitrogen fixation and soil nitrogen was used by SPSS 19.0 (SPSS Inc., Chicago, IL, United States). The factor analysis of nitrogen content, utilization, and traits of forages was performed using Origin Pro 2021 (OriginLab, Northampton, MA, United States). AMOS 21.0 (Amos Development Co., Armonk, NY, United States) was used to analyze the relationship between different variables by using a structural equation model (SEM).

## Results

### Effects of water on nitrogen sources

#### Effect of water on nitrogen fixation and nitrogen transfer

The biological nitrogen fixation rates of M.s and In-M.s increased with increase in the amount of water (−50% < CK < +50%; Table 2). The nitrogen fixation rates of M.s were 8.0, 10.7, and 17.7% in the −50%, CK, and +50% treatment groups, respectively (*P* < 0.05, Table 2). The nitrogen fixation rates of In-M.s were 12.6, 14.9, and 23.0% in the −50%, CK, and +50% treatment groups, respectively (*P* < 0.05, Table 2). Additionally, the nitrogen fixation rate of In-M.s was higher than that of M.s under the same water conditions.

**TABLE 2:** Effect of water on nitrogen fixation and nitrogen transfer.

	Different water level			Change range	
	CK	−50%	+50%	−50%	+50%
In-Pndfa(%)	14.95 ± 0.39b	12.62 ± 0.37c	23.06 ± 1.40a	−15.58 ± 2.61b	54.28 ± 14.95a
Ms-Pndfa(%)	10.71 ± 0.39b	8.04 ± 0.72c	17.74 ± 0.99a	−24.95 ± 8.04b	65.63 ± 10.71a
Pndfl(%)	15.61 ± 0.94b	9.15 ± 0.70c	19.39 ± 0.38a	−41.38 ± 9.15b	24.22 ± 15.61a
%Ndfs(En)	84.39 ± 0.54b	90.85 ± 0.53a	80.61 ± 0.50c	7.65 ± 0.85a	−4.48 ± 0.39b
%Ndfs(Ms)	89.29 ± 0.74b	91.96 ± 0.76a	82.26 ± 0.92c	2.99 ± 0.38a	−7.87 ± 1.53b
%Ndfs(In-Ms)	85.05 ± 0.39b	87.38 ± 0.38a	76.94 ± 0.41c	2.74 ± 0.96a	−9.54 ± 1.29b
%Ndfai	4.74 ± 0.41b	4.98 ± 0.39b	6.46 ± 0.44a	5.00 ± 0.98b	36.30 ± 4.74a
In-Wndfa	0.84 ± 0.05b	0.63 ± 0.05c	3.59 ± 0.07a	−24.67 ± 10.63b	328.32 ± 40.84a
Ms-Wndfa	0.64 ± 0.04b	0.64 ± 0.08b	2.35 ± 0.07a	0.37 ± 0.64b	267.99 ± 50.64a
Wndfl	0.68 ± 0.01b	0.26 ± 0.01c	0.98 ± 0.01a	−61.73 ± 2.21b	43.92 ± 9.20a
NFIR-In	8.42 ± 0.47b	8.05 ± 0.40b	17.42 ± 0.49a	−4.38 ± 8.59b	106.93 ± 12.18a
NFIR-Ms	10.71 ± 0.42b	8.04 ± 0.98c	17.74 ± 0.41a	−24.95 ± 8.04b	65.63 ± 10.71a

In-Pndfa(%), biological nitrogen fixation rate of In-M.s; Ms-Pndfa(%), biological nitrogen fixation rate of M.s; Pndfl(%), the percentage of nitrogen that In-E.n obtained from nitrogen fixed by In-M.s; %Ndfs(En), the percentage of N absorbed by E.n; %Ndfs(Ms), the percentage of N absorbed by M.s; %Ndfs(In-Ms), the percentage of N absorbed by IN-M.s; %Ndfai, the increased rate of nitrogen fixation of In-M.s caused by the transfer of nitrogen fixation products in intercropping; In-Wndfa, biological nitrogen fixation capacity of In-M.s; Ms-Wndfa, biological nitrogen fixation capacity of M.s; Wndfl, the nitrogen transfer amount that In-E.n obtained from nitrogen fixed by In-M.s; NFIR-In, the contribution rate of biological nitrogen fixation to grassland nitrogen yield of In-M.s; NFIR-Ms, the contribution rate of biological nitrogen fixation to grassland nitrogen yield of M.s.

Different lowercase letters in the same column show significant differences (P < 0.05).

The biological nitrogen fixation rate in In-M.s (0.63, 0.84, and 3.59 g/m^2^, respectively, for the −50%, CK, and +50% treatment groups) increased with the increase in the quantity of water. The biological nitrogen fixation of M.s was the highest (2.35 g/m^2^) under the +50% treatment, which was significantly different from that observed under the CK and −50% treatments (*P* < 0.05, Table 2). However, no significant differences were observed between the biological nitrogen fixation rates in the CK and −50% treatments (*P* > 0.05). The change of nitrogen fixation capacity in In-M.s was higher than that of M.s, and the change in the nitrogen fixation rate of In-M.s was more stable than that of M.s under water fluctuations. Therefore, we see that water increase cannot only promote the nitrogen fixation rate of *M. sativa* but also improve its biomass and facilitate further biological nitrogen fixation.

The nitrogen transfer rate and nitrogen transfer amount from nitrogen fixed by In-M.s to In-E.n increased significantly with water increase. Nitrogen transfer was more easily affected by a decrease in water content than by an increase in water content.

The proportion of nitrogen absorbed by In-E.n and In-M.s from the soil decreased with the increase in water content (Table 2). The proportion of nitrogen absorbed by In-E.n from the soil were 90.8, 84.4, and 80.6% in the −50%, CK, and +50% treatment groups, respectively (*P* < 0.05, Table 1). The proportions of nitrogen absorbed by In-M.s from the soil were 87.4, 85.1, and 76.9% in the −50%, CK, and +50% treatment groups, respectively (*P* < 0.05, Table 2). The proportion of nitrogen absorbed by In-E.n from the soil was higher than that by In-M.s under the −50% and +50% treatments (Table 2).

The proportions of nitrogen absorbed by M.s from soil decreased with the increase in water content: 91.9, 89.3, and 82.3% in the −50%, CK, and +50% treatment groups, respectively (*P* < 0.05, Table 2). These results showed that the absorption of nitrogen from soil by M.s was more easily affected by water increase, which is consistent with the result that increased water level was highly conducive to nitrogen fixation. The proportion of nitrogen absorbed by M.s was higher than that by In-M.s, which was related to the understanding that intercropping promotes the nitrogen fixation rate and nitrogen fixation content of In-M.s plants (Table 2).

Intercropping M.s and E.n could improve the nitrogen fixation rate of In-M.s, but the water content would affect the rate of improvement. Nitrogen fixation rate was the highest under the +50% treatment, which was significantly different from that under the −50% and CK treatments, and no significant difference was observed between the −50% and CK treatments (Table 2). The contribution rate of biological nitrogen fixation increased with increase in water content (*P* < 0.05, Table 2). Thus, increased water levels were beneficial to the nitrogen fixation of *M. sativa* and changed the proportion of forage that absorbed nitrogen from different sources.

### Effect of water on soil nitrogen

**FIGURE 3:**
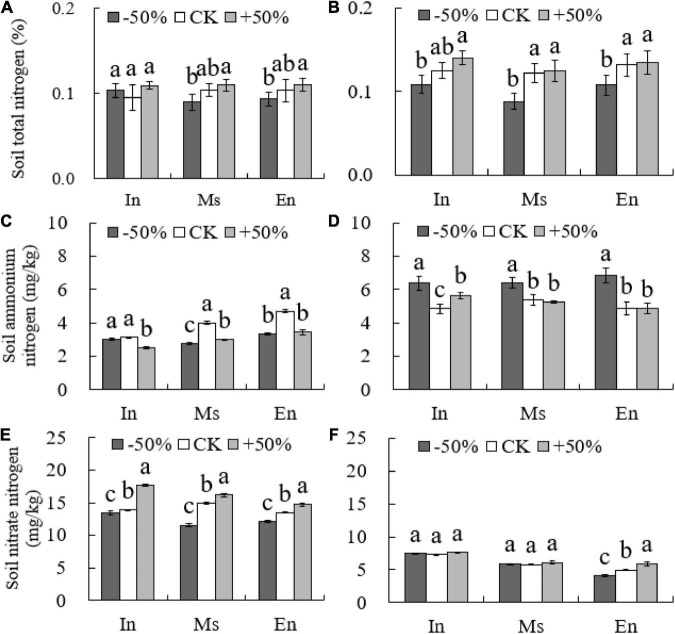
Soil nitrogen content under different water conditions (**A,C,E**: 2015; **B,D,F**: 2016). Different lowercase letters show significant differences under different water levels (*P* < 0.05).

The total soil nitrogen content of M.s and E.n under the −50% treatment was significantly lower than that under the +50% treatment (*P* < 0.05, Figures 3A,B). There was no significant difference in the total soil nitrogen content and soil ammonium nitrogen content between the different types of crop cultivation in the artificial grasslands under the same water conditions (*P* > 0.05, Figures 3A,B).

The levels of soil nitrate-nitrogen increased with an increase in water content (−50% < CK < +50%) for all cropping patterns in 2015. However, no significant difference was observed in the soil nitrate-nitrogen content between the intercropped cultivation and M.s cultivation under different water treatments in 2016 (*P* > 0.05). The content of soil nitrate-nitrogen in E.n plants increased significantly with the increase in water levels (*P* < 0.05, Figure 3), and the content of the intercropped cultivation and M.s cultivation was also significantly higher than that of E.n (*P* < 0.05, Figure 3).

### Relationship between nitrogen fixation, nitrogen transfer, and soil nitrogen

The biological nitrogen fixation rate and amount of *M. sativa* were positively correlated with soil nitrogen (Table 3). In the absence of exogenous nitrogen input, the nitrogen content in the soil did not inhibit the nitrogen fixation of *M. sativa*. The transfer of biologically fixed nitrogen to In-E.n was significantly positively correlated with soil nitrogen (Table 3). The proportions of nitrogen absorbed by In-M.s and In-E.n were significantly correlated with soil nitrogen content (Table 3) but that of M.s was not. Nitrogen was deficient in this area if there was no exogenous nitrogen input, and thus it did not inhibit the biological nitrogen fixation rate. The proportion of nitrogen absorbed from soil by In-M.s, In-E.n, and M.s was inhibited by the content of soil nitrate nitrogen. The higher the soil nitrate-nitrogen content, the lower the proportion of nitrogen absorbed from the soil (Table 3). This was because the soil nitrate-nitrogen content did not only inhibit the biological nitrogen fixation of *M. sativa* but also promoted the fixation proportion and amount.

**TABLE 3:** Regression analysis of nitrogen fixation, nitrogen transfer, and soil nitrogen.

	Total nitrogen	Nitrate nitrogen	Ammonium nitrogen
	0.77**	0.48*	0.12
Ms-Pndfa (%)	−0.04	0.61*	0.18
Pndfl (%)	0.63*	0.75**	0.42*
%Ndfs (E.n)	−0.49*	0.73**	0.50*
%Ndfs (M.s)	−0.58*	0.75**	0.16
%Ndfs (In-M.s)	−0.65**	0.50*	0.13
%Ndfai	0.39	0.17	0.007
In-Wndfa	0.54	0.35	0.044
M.s-Wndfa	0.45*	0.68**	0.061
Wndfl	0.24	0.078	0.005
NFIR-In	0.63*	0.50*	0.16
NFIR-Ms	0.68**	0.57*	0.17

The meaning of abbreviations are same as Table 2.

*Indicates significance at P = 0.05;

**indicates significance at P = 0.01.

### Effect of water level on nitrogen utilization of forage

**FIGURE 4:**
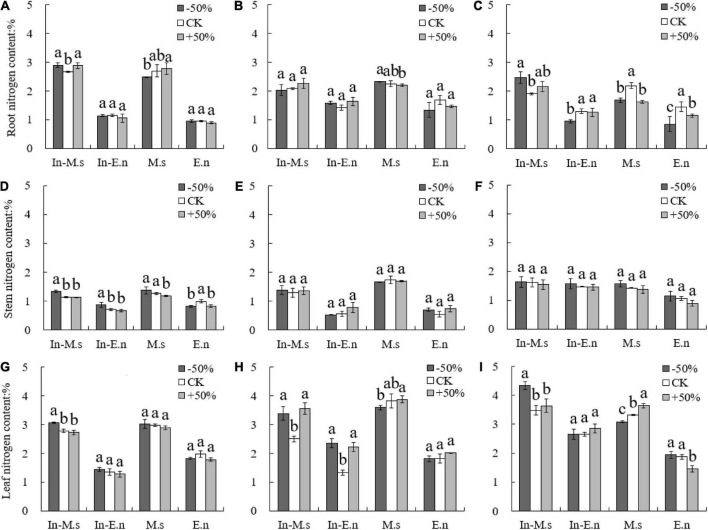
Effect of water on nitrogen content in roots, stems, and leaves of different forages (**A,D,G**: 2015; **B,E,H**: 2016; **C,F,I**: 2017). Different lowercase letters show significant differences under different water levels (*P* < 0.05).

The nitrogen content in roots and leaves was found to be higher than that in stems, and the nitrogen content in roots decreased while that in leaves increased yearly (Figure 4). The nitrogen content in different organs of M.s and In-M.s was significantly higher than that of E.n. However, no significant difference was observed in the nitrogen content of a given species between intercropping and monoculture systems (Figure 4).

**FIGURE 5:**
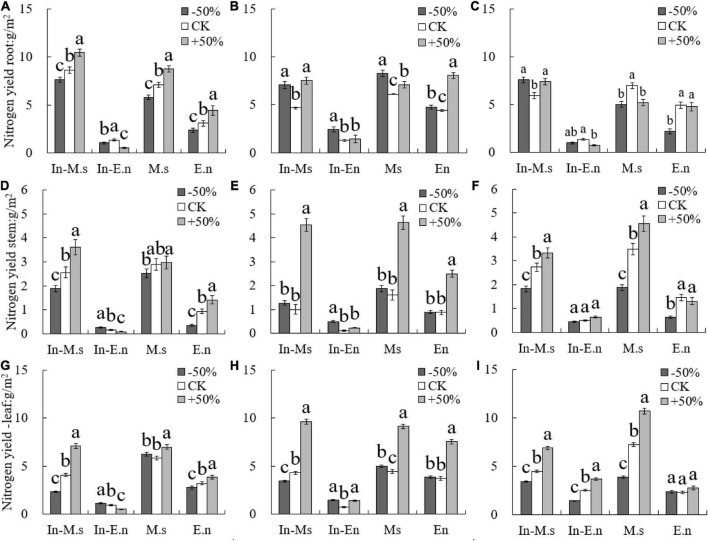
Effect of water on nitrogen yield of different forages (**A,D,G**: 2015; **B,E,H**: 2016; **C,F,I**: 2017). Different lowercase letters show significant differences under different water levels (*P* < 0.05).

The root nitrogen yield of In-M.s and M.s increased with water increase only in 2015 (Figure 5), which may be owing to the root growth of *M. sativa* in the first year of forage planting. However, the root nitrogen yield of In-E.n decreased significantly with water increase (Figure 5), owing to the enhanced competitiveness of In-M.s that inhibited the growth of In-E.n. The nitrogen yield of roots in E.n increased significantly with an increase in water content (Figure 5).

The nitrogen yield of the stem and leaf of In-M.s, M.s, and E.n increased significantly with water increase (Figure 5). The nitrogen yield of the stem and leaf of In-E.n decreased significantly with water increase in 2015 and 2016 but increased in 2017 (Figure 5). This finding was observed because in the early stage of the establishment of the intercropped artificial grassland, water increase was advantageous for In-M.s, weakening the competitiveness of In-E.n (which led to a decrease in the biomass of In-E.n and a decrease in the nitrogen yield of different organs of In-E.n). The nitrogen content of In-E.n was significantly higher in 2017 than in the previous 2 years. In-E.n was able to compete better and increase its absorption of water and nitrogen with time (Figure 5).

**FIGURE 6:**
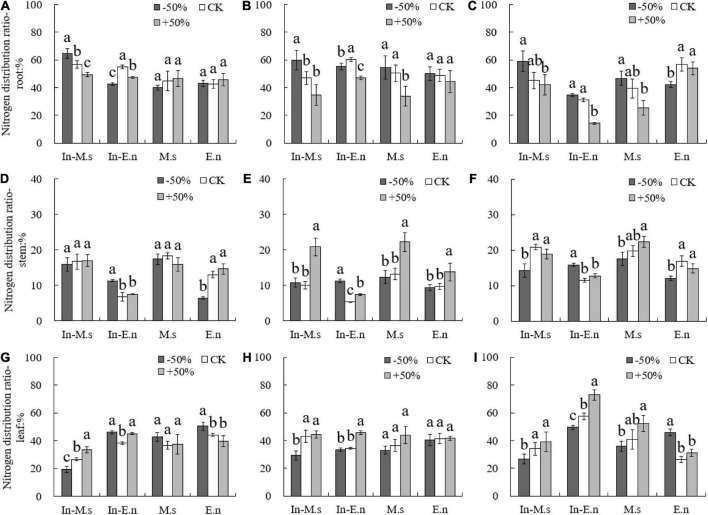
Effect of water on nitrogen distribution in roots, stems, and leaves of different forages (**A,D,G**: 2015; **B,E,H**: 2016; **C,F,I**: 2017). Different lowercase letters show significant differences under different water levels (*P* < 0.05).

The proportion of nitrogen distribution in the roots of In-M.s and M.s decreased significantly with water increase, and water increase was more conducive for the distribution of nitrogen to the aboveground parts of *M. sativa* (Figure 6). In 2017, the proportion of nitrogen distribution in the root of In-E.n decreased and that of E.n increased with water increase (Figure 6). This difference was because water increase promoted the competition between In-M.s and In-E.n, which inhibited growth and reduced the proportion of nitrogen distribution in the root of In-E.n.

**FIGURE 7:**
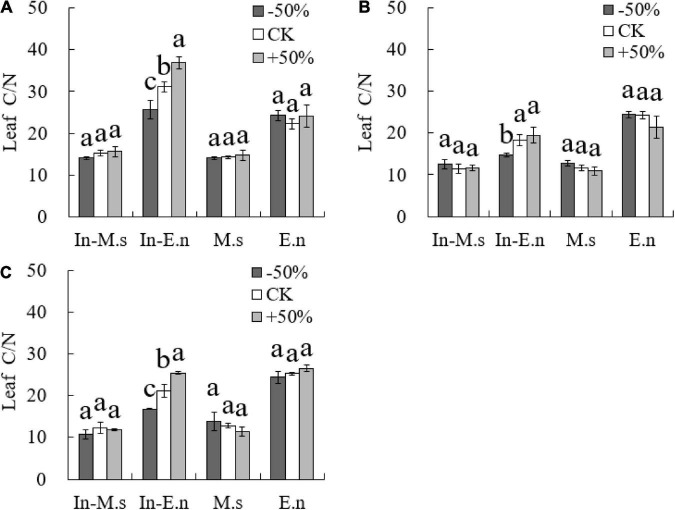
Effects of water on nitrogen use efficiency of forages (**A:** 2015; **B:** 2016; **C:** 2017). Different lowercase letters show significant differences under different water levels (*P* < 0.05).

The NUE of In-E.n increased significantly with water increase (*P* < 0.05, Figure 7), which was related to the water use efficiency of In-E.n. Most studies have shown a negative correlation between NUE and water use efficiency. Therefore, water decrease reduced the NUE of forage.

### Effect of water and soil nitrogen on forage traits

**FIGURE 8:**
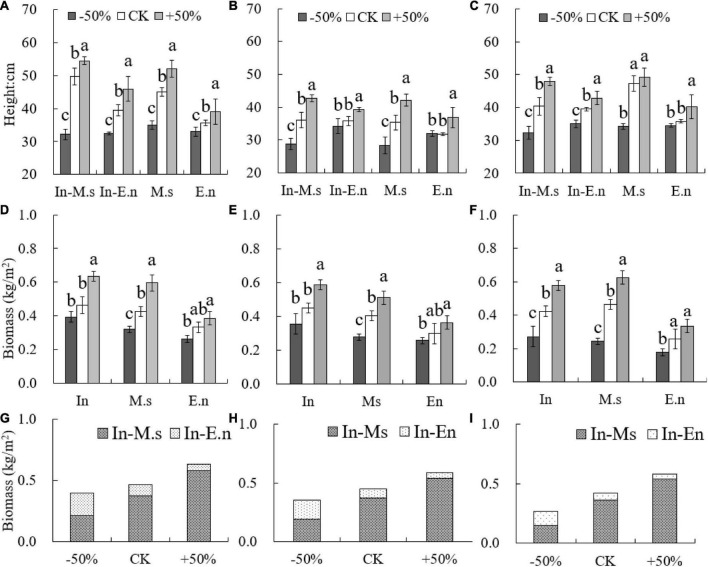
Differences in height and productivity under different water conditions (**A,D,G**: 2015; **B,E,H**: 2016; **C,F,I**: 2017). Different lowercase letters show significant differences under different water levels (*P* < 0.05).

The height of In-E.n was greater than that of E.n under the CK and +50% treatments (Figures 8A,C). The height of *M. sativa* was more sensitive to water decrease, whereas that of *E. nutans* changed more significantly with water increase (Figure 8). *E. nutans* could adapt to the environment better than *M. sativa* when water availability was poor, and water increase was more beneficial to the growth of *M. sativa*. The biomass of M.s was significantly different under the three treatments, and the biomass of *E. nutans* decreased under the −50% treatment but was not significantly different from that under the CK treatment. *E. nutans* was drought-tolerant, and this competitive advantage led to the decrease in biomass of In-M.s under poor water conditions (Figure 8).

**FIGURE 9:**
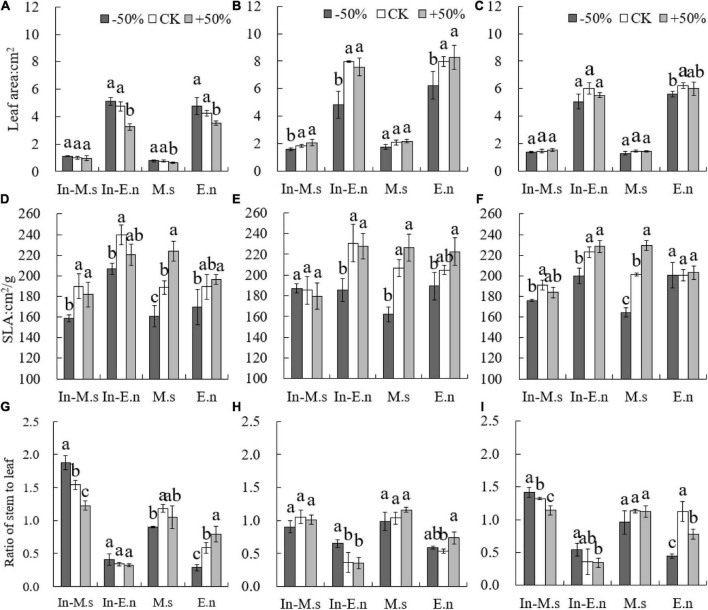
Differences in forage traits under different water conditions (**A,D,G**: 2015; **B,E,H**: 2016; **C,F,I**: 2017). Different lowercase letters show significant differences under different water levels (*P* < 0.05).

The leaf area of *E. nutans* was more significantly affected by water than that of *M. sativa*. The SLA of *M. sativa* and *E. nutans* was significantly low under the −50% treatment (*P* < 0.05) (Figure 9). The SLA of In-M.s was significantly lower than that of M.s under the +50% treatment (*P* < 0.05). The SLA of In-E.n increased significantly compared with that of E.n (*P* < 0.05). Additionally, the stem/leaf ratio of E.n increased significantly and that of In-E.n decreased with water increase (*P* < 0.05). Water increase was beneficial to the leaf biomass of In-E.n (*P* < 0.05). The stem/leaf ratio of In-M.s and M.s showed little difference, but the stem/leaf ratio of In-E.n was significantly lower than that of E.n under the same water conditions. The stem/leaf ratio of *M. sativa* was significantly higher than that of *E. nutans*, but *E. nutans* could obtain more leaf area under the same biomass.

### Relationship between nitrogen utilization and forage traits

**FIGURE 10:**
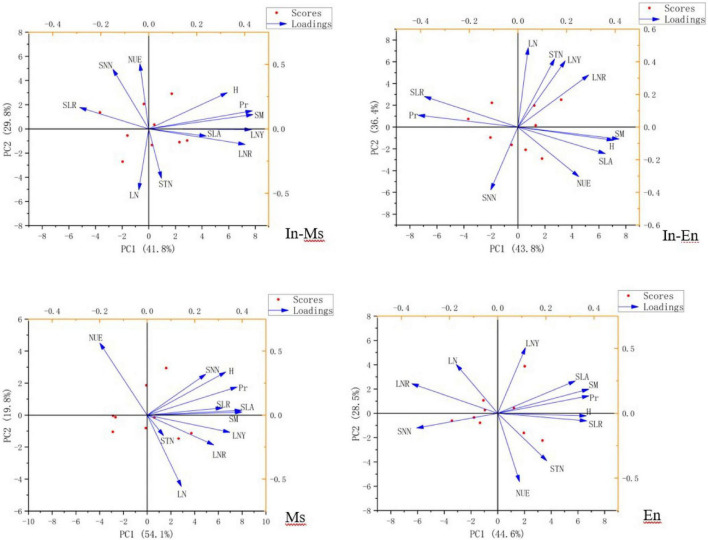
Factor analysis of nitrogen content, utilization, and traits of forages (SM, soil moisture; SNN, soil nitrate-nitrogen; NUE, nitrogen use efficiency; SLA, specific leaf area; H, height; Pr, productivity; SLR, ratio of stem to leaf; LNY, nitrogen yield of leaf; LNR, nitrogen distribution in roots, stems, and leaf; STN, soil total nitrogen content; LN, nitrogen content of leaf).

Soil nitrate-nitrogen, soil total nitrogen content, NUE, and leaf nitrogen content were not conducive to the traits and productivity of In-M.s. Soil water, soil nitrogen content, plant nitrogen content, and NUE were not conducive to the productivity of In-E.n. The competition between In-M.s and In-E.n intensified with water increase, with In-M.s having the absolute advantage. Therefore, water and nitrogen were not conducive to the productivity of In-E.n. Water and nitrogen were beneficial to the productivity and traits of M.s but NUE was not (Figure 10).

**FIGURE 11:**
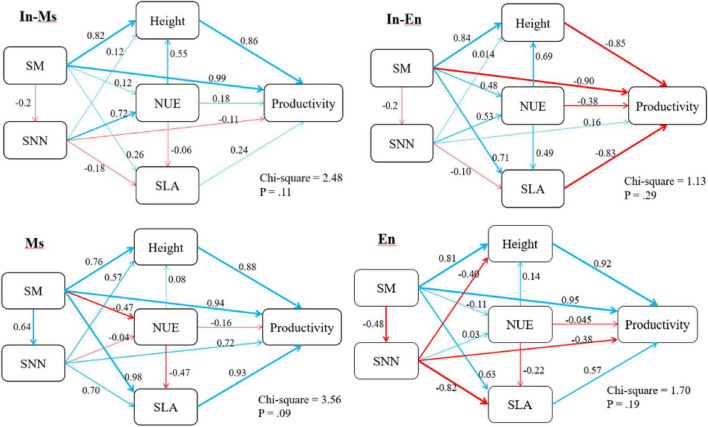
Structural equation model of soil moisture (SM), soil nitrate-nitrogen (SNN), nitrogen use efficiency (NUE), and specific leaf area (SLA).

Soil moisture was significantly conducive to the height and productivity of In-M.s, M.s, and E.n, and the effect of water on productivity was mainly realized through the effect on height. Soil moisture was beneficial to the height of In-E.n but not to its productivity because In-M.s gained a more competitive advantage with an increase in soil moisture. Soil moisture increased the loss of soil nitrate-nitrogen, which was not conducive to the traits of forage in E.n artificial grassland (Figure 11).

## Discussion

### Effects of water on productivity and traits of forages

The relationship between the primary productivity of water and grasslands has been widely studied, and a significant positive correlation has been observed between aboveground primary productivity and annual precipitation in natural (Knapp and Smith, 2001; Bai et al., 2008) and artificial (Zhu et al., 2014; Wang et al., 2015) grasslands. Similar results were obtained in this study, which showed that water increase was beneficial to productivity. However, E.n artificial grassland, although drought-tolerant, did not show an advantage in productivity when the water content was decreased. The productivity in intercropped artificial grassland was higher than that of monocultures when the water level was decreased. In-E.n and In-M.s could ensure more efficient use of water under fluctuating conditions of water levels, thereby increasing productivity.

The functional traits of plants were closely related to growth strategies and resource utilization and could reflect the adaptability to different habitats and environmental changes. SLA of *Leymus chinensis* increased significantly as opposed to that of *E. nutans* with water increase (Tian et al., 2010; Shen, 2016). The results of the present study showed that the SLA of *M. sativa* and *E. nutans* increased with water increase. However, the SLA of In-M.s was less than that of M.s, which may have been due to the competition between In-M.s and In-E.n. The stem/leaf ratio decreased with water increase (Huo et al., 2013). However, some studies suggest that the stem/leaf ratio of *M. sativ*a increases with water increase because the inhibition of water stress on stem growth was greater than that on leaf growth. The stem/leaf ratio of In-M.s and In-E.n was decreased, while that of M.s and E.n increased with a rise in water levels. The results showed that the response of stem/leaf ratio to water varied depending on whether the adopted strategy includes monoculture or intercropped cultivation system. The ratio of stem to leaf in In-M.s and In-E.n decreased with an increase in water content and indicated that the forage quality was better, but the ratios of M.s and E.n were different. In-M.s and In-E.n would put more resources obtained from competition into leaf growth, so that more leaves and leaf area can obtain more amount of light. Interspecific competition between In-M.s and In-E.n changed the response of species traits to water. Water increase not only improved productivity but also improved forage quality in an intercropped artificial grassland.

### Effects of water on nitrogen distribution in soil and plants

Soil water is the carrier of soluble nitrogen, and precipitation is the driving force of soil water movement. Thus, leaching loss and migration intensity of soil nitrogen increased with an increase in water levels. In this study, the content of soil nitrate-nitrogen was significantly different across the three treatments and was the highest under +50% treatment in 2015. This may be because 2015 was the first year of grass planting. The growth of forage roots would have retained the soil water in the shallow layer, thereby mitigating leakage to deeper layers and preventing significant leaching of soil nitrogen. The water content was higher and more conducive to the growth of forage, resulting in higher soil nitrate content. There was no significant difference in the content of soil nitrate-nitrogen under different water treatments in 2016, which might be because the growth of grass roots slowed down in the second year, and the effects of soil water migration on the surface layer caused by root water uptake and soil water leakage to the deep layer were the same among different water levels, showing that differences in the water level did not have a significant impact on soil nitrate-nitrogen content.

The content of soil nitrogen differed for different forages. Under the same water condition, there was no significant difference in soil total nitrogen and ammonium nitrogen content among M.s, E.n, and In crops, but the soil nitrate-nitrogen content of M.s and In was significantly higher than that of E.n. Gramineous grasses consume a lot of nitrogen in the growing season, leading to severe nitrogen deficiency in the absence of an external nitrogen supplement. M.s could balance the consumption and accumulation of soil nitrogen and even increase soil nitrogen content because of its biological nitrogen fixation capacity (Gao et al., 2015). Although no significant difference was observed in intercropping of legumes compared to monocropping, it is claimed that intercropping would be more conducive to the accumulation of soil nitrogen compared to monoculture because gramineous grasses absorb soil nitrate-nitrogen and reduce the inhibition of biological nitrogen fixation of legumes in intercropped cultivation (Karpenstein-Machan and Stuelpnagel, 2000; Peoples et al., 2015; Li et al., 2020).

The leaf is the main organ of photosynthesis and the key source of nutrients, including nitrogen (Lopez-Bellido et al., 2004). Water deficiency, that is, drought stress, can reduce the nitrogen content in leaves (Sinclair et al., 2000). However, some studies have shown that nitrogen transport to leaves does not increase significantly under drought stress (Banedjschafie et al., 2008; Xu et al., 2010), possibly owing to a change in nitrogen transport under drought stress (which is influenced by many factors, including the difference between species and degree of drought stress). The results showed that the nitrogen content of different organs followed the order leaf > root > stem. The nitrogen content of roots decreased and that of leaves increased in the second year of grass planting; this may have been because of higher metabolic activity (and hence higher requirement of nitrogen) of the roots than the aboveground parts in the first year of grass planting. The nitrogen content of stems was not significantly different between the 2 years because the stem is the organ that transports nutrients. However, other studies have shown that the stem is an important nitrogen source in the process of plant growth, and its nitrogen content was second only to the leaf (Xu et al., 2010). Our results showed that the nitrogen content of stems was lower than that of the roots and leaves. Forages only use vegetative organs; therefore, to improve their adaptability and competitiveness, forages store nitrogen in their main growing parts (Yu et al., 2006; Grechi et al., 2007).

### Effects of water on biological nitrogen fixation and nitrogen transfer

The productivity advantage of intercropping legumes and Gramineae grasses is closely related to the efficient use of nitrogen (Karpenstein-Machan and Stuelpnagel, 2000). Legumes generally meet their own nutritional needs through biological nitrogen fixation and reduce the competitive absorption of soil nitrogen, while Gramineae grasses use soil nitrogen as their main source of nitrogen, and their competitive ability to absorb soil nitrogen is greater than that of legumes (Li et al., 2001). In intercropped artificial grasslands, biological nitrogen fixation by legumes can also provide a certain amount of nitrogen for Gramineae grasses, thus guaranteeing that an intercropped artificial grassland has sufficient nitrogen. The biological nitrogen fixation rate of *M. sativa* when intercropped with *Elymus sibiricus* was 19.5% higher than that of a monoculture under the same conditions. Our results also showed that the nitrogen fixation rate of In-M.s and M.s increased with water increase and that the nitrogen fixation rate of In-M.s was higher than that of M.s under the same water conditions. Our results were consistent with those of other studies showing that the nitrogen fixation efficiency of legumes in intercropped cultivation was higher than when grown as a monoculture (Corre-Hellou et al., 2006; Schipanski and Drinkwater, 2012). This is because soil nitrogen directly affects the nitrogen fixation of legumes, which is affected by the competition between legumes and Gramineae grasses in intercropping conditions (Schipanski and Drinkwater, 2012) where the inhibition of soil nitrogen on nitrogenase activity is reduced (Hauggaard-Nielsen et al., 2003).

Environmental conditions also affect the nitrogen fixation capacity of legumes. Water has a major impact on the biological nitrogen fixation of legumes in the typical steppe of Inner Mongolia where there is less precipitation. The nitrogen fixation of an artificial grassland with intercropping of *Lolium perenne* and *Trifolium repens* was 90 kg N ha^–1^ yr^–1^ under drought conditions and 240 kg N ha^–1^ yr^–1^ under humid conditions. In the present study, the nitrogen fixation of *M. sativa* decreased under drought conditions. This is because drought can affect the formation of nodules (Pons et al., 2007), reduce the number of nodules, and inhibit the activity of nodule nitrogenase (Mnasri et al., 2007). Therefore, drought can limit the nitrogen fixation capacity of legumes.

Soil nitrogen content also affects the biological nitrogen fixation. Plants preferentially absorb inorganic soil nitrogen, particularly nitrate nitrogen, compared with fixed nitrogen. Therefore, high soil inorganic nitrogen content reduces the nitrogen fixation capacity of legumes. However, in our study, nitrogen fixation and nitrogen transfer were significantly positively correlated with soil nitrate nitrogen. This may be owing to the lack of added nitrogen in the artificial grassland, which meant that the inorganic soil nitrogen content was insufficient to limit the nitrogen fixation capacity of *M. sativa*. Although In-M.s and In-E.n could improve the nitrogen fixation rate of legumes, water conditions would also influence the same. The better the water condition, the more obvious the rate of improvement. This is because under better water conditions, the growth of In-E.n and In-M.s artificial grasslands is better. In-E.n would absorb more nitrogen to meet their own growth needs, thus reducing the inhibition of soil nitrogen on biological nitrogen fixation of In-M.s. Therefore, better water conditions facilitate greater biological nitrogen fixation in In-M.s.

### Relationship between water and nitrogen use

Many studies on intercropped legumes and Gramineae grasses, such as intercropped *Pisum sativum* and *Zea mays* (Li et al., 2011), *P. sativum* and *Hordeum vulgare* (Hauggaard-Nielsen et al., 2003), and *P. sativum* and *Triticum aestivum* (Bedoussac and Justes, 2010), have shown that such intercropping is conducive to nitrogen absorption and accumulation. However, some other studies have shown that such intercropping did not promote nitrogen absorption by Gramineae grasses. For instance, the nitrogen accumulation of *Saccharum officinarum* did not significantly improve under *S. officinarum* and *Glycine max* intercropping conditions (Yang et al., 2011), but the nitrogen absorption of these plants was higher under intercropping conditions than under monocultures (Yang et al., 2013). Our results showed that the nitrogen yield of forage stems and leaves increased with water increase in an intercropped grassland and M.s monoculture grassland, resulting in higher soil nitrogen output. Compared with M.s and E.n monocultures, although the intercropped grassland showed higher and more stable productivity with insufficient water, the soil nitrogen content decreased owing to the lower nitrogen fixation capacity of In-M.s, resulting in insufficient nitrogen content to compensate for the nitrogen output caused by mowing. The intercropped artificial grassland not only obtained higher productivity and nitrogen yield but also maintained the soil nitrogen level when the water levels were insufficient. Therefore, if precipitation fluctuates, the proportion of gramineous grasses in artificial grassland should be increased for better adaption to the lower levels of water when precipitation is less. The proportion of leguminous forage, which supports higher productivity, promotes the accumulation of soil nitrogen, and alleviates the limitation of nitrogen on the productivity of artificial grasslands, should be increased when precipitation is sufficient.

## Conclusion

In this study, we analyzed the effects of different levels of water on the relationship between nitrogen sources, nitrogen utilization, and forage traits in artificial grasslands composed of monocultures of *M. sativa* and *E. nutans* and intercropped *M. sativa* and *E. nutans* crops. The main results of the study are as follows:

(1) *E. nutans* could adapt to the environment better than *M. sativa* when water levels were poor. The biomass obtained by intercropping *M. sativa* and *E. nutans* crops showed no significant advantage over that of monoculture M.s under +50% treatment but decreased to less than that of M.s under −50% treatment. The water levels significantly affected the leaf area and SLA of *E. nutans* but had little effect on those of *M. sativa*.
(2) E.n obtained all its nitrogen from the soil. The nitrogen fixation rate of *M. sativa* was higher, and the proportion of nitrogen absorbed by *M. sativa* from soil decreased under +50% compared to that under CK treatment. In-M.s could obtain more nitrogen by biological nitrogen fixation than M.s under the same water level condition. The proportion of nitrogen absorbed from soil by In-E.n decreased with the increase of water content.
(3) Soil moisture increased the loss of soil nitrate-nitrogen, and the content of soil nitrate-nitrogen was not conducive to the traits of E.n. Under different planting patterns, different forages utilized water and soil differently because of varied forage growth and traits that further influenced the effects of soil water and soil nitrogen on the growth of the forages. *M. sativa* in intercropping reduced the utilization and loss of soil nitrogen, and intercropping of *M. sativa* and *E. nutans* could result in higher productivity as well as improve the biological nitrogen fixation than the monocultures of *M. sativa* or. *E. nutans*.

## Data Availability

The data that support the findings of this study are available from the ZW, upon reasonable request.
